# Nanoliter-Fabricated Paper-Based Colorimetric Lateral Flow Strip for Urea Detection

**DOI:** 10.3390/bios15100688

**Published:** 2025-10-11

**Authors:** Supatinee Kongkaew, Suparat Cotchim, Warakorn Limbut

**Affiliations:** 1 Division of Health and Applied Sciences, Faculty of Science, Prince of Songkla University, Hat Yai, Songkhla 90110, Thailand; supatinee.k@psu.ac.th (S.K.); suparat.c@psu.ac.th (S.C.); 2 Center of Excellence for Trace Analysis and Biosensor, Prince of Songkla University, Hat Yai, Songkhla 90110, Thailand; 3 Center of Excellence for Innovation in Chemistry, Faculty of Science, Prince of Songkla University, Hat Yai, Songkhla 90110, Thailand; 4 Forensic Science Innovation and Service Center, Prince of Songkla University, Hat Yai, Songkhla 90110, Thailand

**Keywords:** miniaturization, lateral flow strip, urea, colorimetric, biosensor

## Abstract

A nanoliter-scale fabrication method was applied to construct a colorimetric lateral flow strip for urea detection (Urea-CLFS). The device involves two main papers: a nitrocellulose membrane (NC-Mb) for urease enzyme immobilization and chromatography paper (CH-PP) containing a phenol red indicator. Urea-CLFS is a tool for detecting urea that is based on enzyme catalysis and the change in color of phenol red when urea is present. The Urea-CLFS fabrication was made possible by the minimal amount of nanoliters used in reagent consumption. The use of small arrays of phenol red dots provides a higher response result compared to single dots applied on CH-PP. To find the most effective design, it analyzed how urease was aligned on NC-Mb horizontally and vertically. According to our findings, the vertical alignment of the urease enzyme on NC-Mb leads to a prolonged reaction time, which leads to higher product production. The optimization process included optimizing various parameters, including the layer number of phenol red on CH-PP, phenol red concentration, urease concentration, reaction time, and sample volume. Under optimal conditions, the Urea-CLFS provided a linear range of 0.25–8.0 mmol L^−1^ with an LOD of 0.34 mmol L^−1^, which is sufficient for human health diagnostics. The accuracy of the Urea-CLFS was demonstrated by the recovery of the human urine sample between 95 ± 3% and 103 ± 3% (n = 3).

## 1. Introduction

Miniaturization of chemical/bio analysis has become a prominent trend in modern analytical chemistry. Miniaturized devices have both the ability to analyze small samples and the ability to reduce reagent consumption during fabrication, making them the main advantages. Microdroplets are commonly categorized as liquids with small volumes, typically in the range of femto/picoliter to nanoliters, which are suitable for miniaturized devices. Microdroplet chemistry can aid laboratories in preserving valuable compounds, decreasing sample volumes and waste streams, and accelerating analyses [1]. The production of analytical instruments and the implementation of analysis are facilitated by miniaturized devices that have a low environmental impact and reduce economic costs [2]. Microdroplets have been used in different fields over the years, particularly microfluidics. Droplet creation, characterization (measurement of shape, size, and volume), and manipulation are all aimed at producing droplets reliably, transporting them once produced, and using them as sensors [1]. Despite the advancements in microdroplet-based research and applications, there are still challenges that need to be explored further in these scenarios.

Paper-based material has been employed for immobilized biomolecules in many bioassays for a long time. Paper is a porous membrane that can function at a small volume (µL-nL) and deliver a sample via capillary action [3]. Enzyme-embedded paper-based analytical devices (PADs) have recently gained much attention due to their exceptional benefits. Enzymes are complex macromolecules composed of proteins and biological catalysts that possess high catalytic ability and specificity [4]. Based on an enzyme biosensor, the biosensor’s response can be amplified, a wide variety of commercial enzymes can be utilized, reactions can be fast, and real-time monitoring and analysis can be performed [5,6]. The combination of enzymes and PADs results in significant improvements in analytical performance and excellent chemical and storage stability. Immobilization of enzymes on PADs can be achieved via different methods, including covalent binding, physical adsorption, and physical entrapment [7]. Additionally, PADs have the capability to integrate multiple functions, including sample collection, transportation, and analysis, in small areas. PADs are typically surrounded by hydrophobic areas and paper zones that provide hydrophilic areas. The paper can be patterned for the hydrophobic barrier or shaped and cut to create well-defined hydrophobic and hydrophilic areas [8]. Different methods can be used to fabricate the hydrophobic part of the PAD, including wax printing, photolithography, inkjet printing, and laser printing [9]. Over the past few decades, diagnostics have become more popular with the use of lateral flow strips (LFSs), a type of PAD that is easy to use. For LFS fabrication, there is no need for a hydrophobic/hydrophilic pattern on the paper since strips are cut to form a pattern platform, and the liquid will flow to the test zone through capillary action. Nitrocellulose is a commonly used substance in LFSs, and other paper-based materials can also be included in the device. Nitrocellulose (NC) stands out in terms of its high protein binding affinity compared to other papers. NC is made of nitrate cellulose by substituting hydroxyl groups with nitrate groups [10]. It facilitates the efficient immobilization of capture agents for enhanced detection sensitivity [11]. By using only paper, this approach becomes more accessible to the end-user, which is advantageous because it is cheaper, lighter, simpler, and easier to transport and store. Additionally, it is suitable for multiple detection techniques, such as colorimetric, fluorescent, and chemiluminescent readouts. Numerous studies have exemplified the use of nitrocellulose membranes to immobilize enzymes on paper-based devices for biosensor applications [12,13]. However, the integration of nitrocellulose and chromatography paper to improve the performance of colorimetric sensors has not been well documented.

Urea is a nitrogenous waste product of metabolism, which is crucial in biological systems. The main way humans remove waste nitrogen is through protein and amino acid metabolism, which involves the synthesis of urea. This process is carried out in the liver through the urea cycle. The kidneys remove urea from the blood and excrete it into urine [14]. Urea can also be found in other types of body fluids, including saliva, and blood. Human blood has a normal range of urea concentration between 2.6 and 6.5 mmol L^−1^ [14], but it can vary significantly in human urine, leading to a 50-fold increase. Small-volume diagnostics are facilitated by the concentration of urine (275–409 mmol L^−1^) [15], which is the preferred fluid for POC diagnosis. Compared to blood and saliva, it has a collection protocol that is both simple and noninvasive [15]. Additionally, urea is an essential biomarker indicator for biomedical and clinical applications. The presence of abnormal levels of urea is often a sign of different metabolic disorders, such as liver and renal disease, heart failure, or dietary issues [14]. Therefore, the development of portable urea tests that can reduce sample requirements, reduce waste, and require no pretreatment is crucial for diagnosing human health.

Different advanced analytical techniques for urea detection devices can be analyzed such as nuclear magnetic resonance, spectroscopy, chromatography, and electrochemical methods. However, the analysis of most conventional techniques takes time, as sample preparation, costly equipment, and specialized personnel are necessary. Colorimetric stands out against other techniques due to its simplicity, low cost, real-time response, minimal equipment requirement, and visual quantification. Using the urease enzyme, the device is more specific. The colorimetric lateral flow strip (CLFS) is highly favored by end users, as it can be performed by untrained individuals and determined quickly with the naked eye or smartphone, without the need for expensive equipment.

In this work, a paper-based analytical device using a horizontal flow or colorimetric lateral flow strip (CLFS) was developed for the detection of urea. The detection is achieved by introducing ammonia through nitrocellulose paper and causing a colorimetric change in the pH indicator in the chromatography paper’s detection zone. The pH-responsive dye changes color distinctively after reacting with urease and urea on the paper strip, which indicates the presence of urea. Urea-CLFS’s capabilities could be improved by using two different papers for different functions. The separated PADs between the catalyst and detection PADs would improve stability and reduce interference effects. The article demonstrates the use of microdroplets with a small volume (nanoliters) to enhance intensity response and reduce reagent consumption at the same time as detecting the target analyte. Various parameters were comprehensively studied in order to achieve the best Urea-CLFS under horizontal flow conditions. The accuracy, precision, and real sample analysis in human urine were investigated.

## 2. Experimental

### 2.1. Reagents

Urease from *Canavalia ensiformis* (Jack bean) type III 15,000–50,000 units/g solid was obtained from Sigma-Aldrich (Steinheim, Germany). Urea, ethylenediaminetetraacetic acid (EDTA), glycerol, sodium chloride, glucose and creatinine were also sourced from Sigma-Aldrich (Steinheim, Germany). Tris hydrochloride was obtained from LOBA Chemie (Mumbai, India). Phenol red and sodium hypophosphite monohydrate were procured from AJAX FineChem (New South Wales, Australia). Disodium hydrogen phosphate was sourced from Kemaus (New South Wales, Australia). Magnesium sulfate and calcium chloride were obtained from Grammaco (Nonthaburi, Thailand). Hi-Flow^TM^ Plus nitrocellulose membrane (NC-Mb) HFC13502 was procured from Merck Millipore, Darmstadt, Germany (composite thickness: 182 µm, capillary flow: 133 s, width: 2.5 cm). Chromatography paper (CH-PP) was obtained from Whatman 1 (thickness: 180 µm, flow rate 130 mm 30 min^−1^) was obtained from Sigma-Aldrich (Steinheim, Germany).

### 2.2. Solution

A standard stock solution of urea was prepared monthly by dissolving the powder in DI water. The working standards were prepared weekly from the stock solution in the range of 2.5 to 30 mmol L^−1^. A 0.5 mmol L^−1^ sodium phosphate buffer solution (pH 6.6) was prepared with NaH_2_PO_4_ and Na_2_HPO_4_. The urease enzyme solution of 5000 U mL^−1^ was prepared by dissolving the suitable amount of the lyophilized powder in 0.5 mmol L^−1^ sodium phosphate buffer (pH 6.6) containing 1 mmol L^−1^ EDTA, 100 mmol L^−1^ Tris, and 0.5 mol L^−1^ glycerol. The color reagent, 0.1% w/v of PR solution, was prepared by dissolving 5.0 mg of PR powder in 5.0 mL of sodium phosphate buffer solution (pH 6.6).

### 2.3. Design and Development of Urea-CLFS

**Figure 1:**
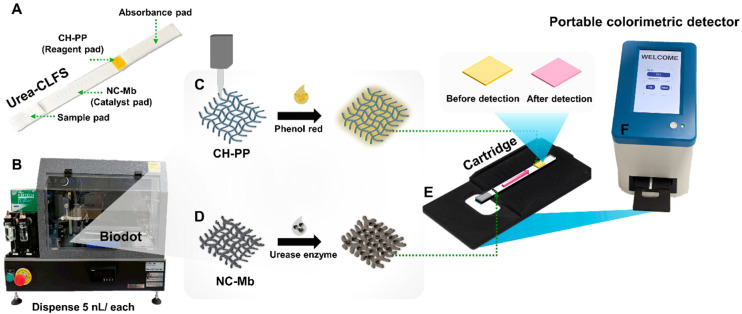
Schematic overview of the fabrication and operation of the Urea-CLFS. (**A**) Layout of the complete test strip including sample, catalyst, reagent, and absorbent pads. (**B**) Biodot system used to dispense 5 nL microdroplets of reagents. (**C**) Preparation of the reagent pad by depositing phenol red onto CH-PP. (**D**) Urease immobilization onto NC-Mb to form the catalyst pad. (**E**) Assembly of the test strip onto a cartridge for analysis. (**F**) Colorimetric detection performed using a portable reader.

Figure 1A displays the compositions of each urea-CLFS that consists of four components. They are a sample pad, an NC-Mb immobilized with urease enzyme (catalyst PAD), the detection PAD (CH-PP), and an absorbance pad. The small nanovolume of reagent/enzyme was dropped using a BioDot device (Figure 1B), in appropriate conditions. The catalyst PAD was made by dispensing a small amount of urease enzyme into NC-Mb during the design alignment (Figure 1D). At the start, urease was designed to arrange 10 dots (5 nL each) on NC-Mb in two rows, producing a total volume of 5 × 20 = 100 nL. For reagent/detection PADs, start by creating a square shape with a size of 3 × 4 mm (4 points) to be a hydrophobic/hydrophilic part on CH-PP using a heat transfer printing device. The indicator solution, phenol red, was used to fill the square with a line array that has 10 dots (5 nL each) for five columns using a BioDot device (Figure 1C). Both papers were dried in an oven at 35 °C for 10 min. The NC-Mb was placed on the cartridge, and the CH-PP was placed at the end of the NC-Mb (Figure 1E). The detection of color products was performed using a portable colorimetric device (Figure 1F).

### 2.4. Detection Procedure and Image Processing

A total of 12 µL of aqueous urea solution was applied to the end track of Urea-CLFS. By hydrolyzing the urea in the aqueous solution, urease produced ammonia and carbon dioxide. The ammonia will turn into ammonium and raise the pH of the solution. The product will be transferred to the reagent PAD and its color will be altered. The color of the product was evaluated by the portable RapidScan ST5-D (Pacific Image Electronic, Taiwan) using Rapid Test View Pro (ST5-D) and RTV_Ethernet software (RTV1.15_b0452-1/ERTV1.37 Build 0000) in the whole work. The profile wizard was set by using the Light source of Epi White, the image method of excellent mode, and the integration method of mean. The detection area was set at 50 × 100 (width × height). RapidScan has four image spectrum settings to choose from: RGB is the color mode, R is for the red channel spectrum, G is for the green channel spectrum, and B is for the blue channel spectrum. They are all single modes, and the intensity achieved in this work is obtained by calculating the grey level of the range of interest (ROI) in each color mode. This intensity was obtained by selecting the integration method “means”, which is recommended in the user manual for biochemistry tests. The intensity of the color products in the four modes, R, G, B, and RGB, was detected in the first two experiments. Afterward, G and RGB were evaluated solely in analytical analysis because they have high sensitivity. The catalytic activity of the enzyme in the oxidation reaction, as well as other parameters, has a significant impact on the response of the paper sensor in order to measure ammonia optimally. The efficiency of the sensor that has been made can be improved through optimization of factors such as the concentration of the reagent, the concentration of enzymes, the volume of the sample, and the reaction time.

## 3. Results and Discussion

### 3.1. Detection Principle of the Test

Enzymatic urea detection has been achieved by a colorimetric lateral flow strip. Through catalysis and conversion, the enzyme urease transforms urea into CO_2_ and NH_3_. The combination of NH_3_ and water produces NH_4_OH, which results in an increase in the environment’s pH. The higher concentration of urea in samples leads to a greater increase in the pH. Phenol red is utilized as a dye to show pH levels. This compound is zwitterionic in nature, having both a negative charge from the sulfate group and a positive charge from the ketone. The loss of hydrogen from the hydroxyl group in the environment occurs when the pH increases, resulting in a pink color. Therefore, a higher level of urea will induce a greater intensity of the color product, which can be used for quantitative analysis of urea.

### 3.2. Nanodots Device

**Figure 2:**
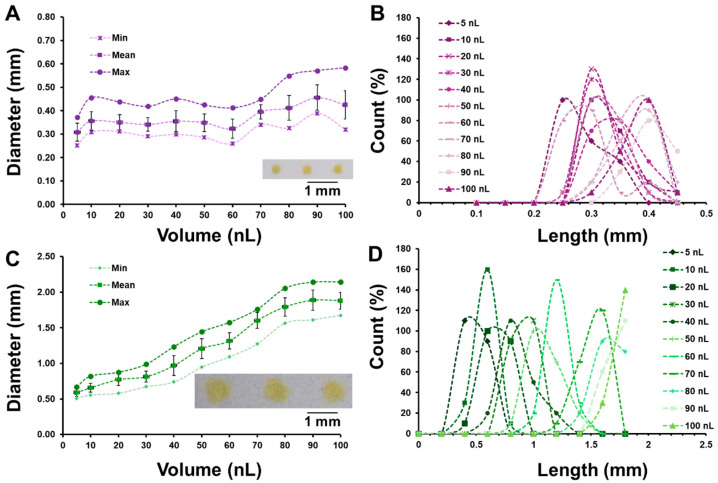
Droplet diameter measured at various volumes and open-time settings, deposited using the Biodot device on (**A**) NC-Mb and (**C**) CH-PP. Size distribution profiles of the droplets are shown in (**B**) for NC-Mb and (**D**) for CH-PP.

This study investigates how volume and appropriate open time affect the diameter length of phenol red reagent dispensed onto paper in order to understand the process of nanodot device work. Different volumes of the sample were examined: 5, 10, 20, 30, 40, 50, 60, 70, 80, 90, and 100 nL. The open time for dispensing was chosen according to the BioJet open time reference chart. The substrates for this study were CH-PP and NC-Mb. The open time for each volume is different depending on the volume, as shown in Table S1. Each volume was performed with 10 drops. ImageJ version 1.53e was employed to measure the size of the reacted drop on both papers in both horizontal and vertical dimensions (10 samples each). Average droplet diameters and distribution patterns for both paper substrates across the tested volumes (n = 20) are shown in Figure 2A–D. NC-Mb reported a minor increase in drop size from 0.37 to 0.58 mm as the sample volume was raised from 5 to 100 nL. The digital image in Figure 2A (inset) shows that NC-Mb has a diameter length of phenol red of 0.31 ± 0.04 mm at 5 nL. The CH-PP substrate can diffuse reagents on paper with a volume of 5 to 100 nL dispensed and have a diameter length of 0.7 to 2.1 mm. Figure 2C (inset) shows that the diameter length of phenol red at 5 nL is 0.59 ± 0.05 mm. As illustrated in Figure 2B,D, the size distribution of NC-Mb was less compared to CH-PP. These differences in droplet diameter and dispersion behavior can be attributed to the intrinsic structural and chemical properties of each paper substrate. CH-PP, composed of cellulose fibers, exhibits a more porous and loosely packed network that promotes greater lateral diffusion of aqueous reagents. This porous morphology allows the liquid to wick and spread over a larger area, resulting in larger and more variable droplet diameters. In contrast, NC-Mb contains nitrocellulose with a denser fiber matrix and smaller pore size, which limits lateral flow. Furthermore, the presence of nitrate functional groups on the NC-Mb surface increases its hydrophobicity, thereby restricting the spread of aqueous phenol red droplets. These combined effects contribute to the smaller and more consistent droplet diameters observed on NC-Mb compared to CH-PP. In this task, CH-PP was used to hold the reagent indicator (phenol red), and NC-Mb was used to bind the urease enzyme.

### 3.3. Morphology Characterization

**Figure 3:**
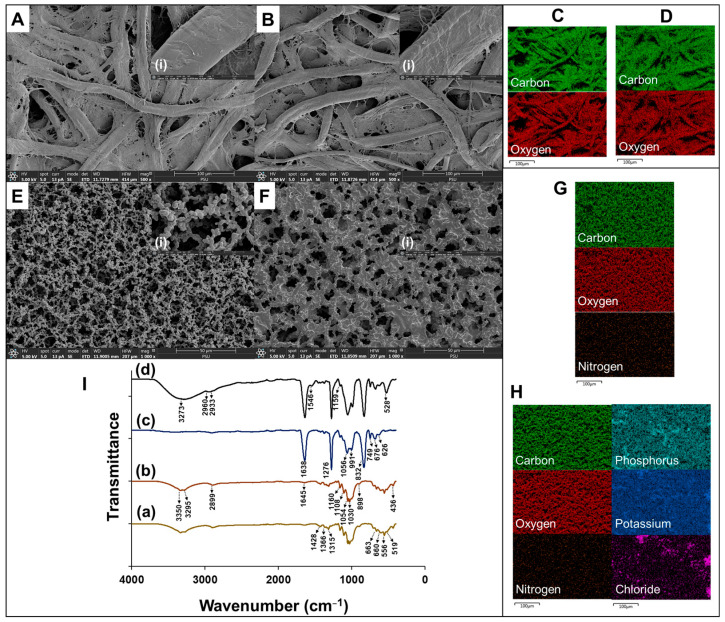
SEM images of CH-PP (**A**) before and (**B**) after application of phenol red reagent on CH-PP, shown at both low and (i) high magnification. EDX mapping of CH-PP substrate (**C**) before and (**D**) after application of phenol red reagent on CH-PP. SEM images of NC-Mb (**E**) before and (**F**) after urease immobilization, shown at both low and (i) high magnification. EDX mapping of NC-Mb substrate (**G**) before and (**H**) after urease immobilization. (**I**) FTIR spectra of CH-PP (**a**) before and (**b**) after application of phenol red reagent and NC-Mb (**c**) before and (**d**) after urease immobilization.

The morphology of CH-PP was visualized using SEM before and after applying the indicator reagent (Figure 3A,B). The SEM images show that both have fibrous structures that are typical of cellulose fibers and are randomly arranged. CH-PP exhibits a smooth microfibers surface typical of cellulose-based materials. CH-PP’s morphology remained unchanged after the indicator reagent was applied. EDX mapping reveals carbon and oxygen, which are the primary components of CH-PP (Figure 3C,D). The EDX mapping after the indicator was applied on CH-PP provides similar elements of carbon and oxygen, since those are the main elements in the phenol red structure as well. The absence of sulfur signals may result from the low emission intensity of sulfur under the operating beam conditions (Figure 3D). The morphology of NC-Mb was visualized using SEM before and after incubation with the urease enzyme (Figure 3E,F). NC-Mb exhibits a consistent network of randomly oriented fibers forming interconnected porous channels. The EDX image shows a homogeneous distribution of carbon, oxygen, and nitrogen elements on paper, which are the main composition of NC-Mb (Figure 3G). The addition of nitrogen in the original NC-Mb is a result of substituting the hydroxyl group with the nitrate group, which is the primary difference between cellulose paper and nitrocellulose paper [10]. This structure is particularly suited for protein adsorption, as it can hold the large protein molecules in the polymer matrix’s high surface area. The possibility of protein adsorption is expected to be facilitated by the smaller pores and large surface areas [11,16]. The fibers thickened considerably after being incubated with urease enzyme, as shown in Figure 3F. Furthermore, EDX shown additionally contained potassium, phosphorus, and chloride elements, as demonstrated in the images (Figure 3H). This result indicates that the urease enzyme was deposited on the surface of NC-Mb.

Identifying their composition was achieved by performing FTIR spectra of the individual pads. Characteristic peaks of CH-PP were presented in Figure 3I(a,b). The absorption peaks at 3350–3295 cm^−1^ were attributed to the -OH stretch vibration, while 2899 cm^−1^ is attributed to C-H stretching vibration. The peak at 1645 cm^−1^ is attributed to the O-H vibration of water. At 1060 cm^−1^, the absorption peak can be attributed to C-O-C vibrating [17]. The peak of 1315 cm^−1^ is attributed to the C-O stretching, while 1160 cm^−1^ C-O-C is attributed to asymmetric stretching. Absorbance peaks at 1030 and 1054 cm^−1^ are attributed to aliphatic C–O stretching and C-O stretching. The peak at 898 cm^−1^ is attributed to the C-H rocking vibrations from the cellulose. These peaks are referred to as the cellulose form of the carbohydrate [18]. The C-H_2_ deformation vibration and C-H groups in cellulose cause the tiny peak located at 1428 and 1366 cm^−1^ to appear. At about 663 and 660 cm^−1^, there are small peaks that indicate the C-OH bending [19]. CH-PP’s structure was unaffected by the indicator since its spectra remains unchanged after application (Figure 3I(a,b)). NC-Mb (Figure 3I(c,d)) showed sharp and strong peaks of about 1638 and 1276 cm^−1^, which are attributed to the asymmetrical and symmetrical stretching vibrations of -NO_2_. The peak located between 990 and 1065 cm^−1^ was attributed to the aliphatic structure of polysaccharides. -NO stretching is responsible for the peak found at 832 cm^−1^ [20]. At 749 cm^−1^, the absorbance is thought to be due to the aromatic ring of the indole group [21]. The structure of NC-Mb is consistent with the result, which enhances the ability of biomolecules to bind to NC-Mb. The NO_2_-functional group’s strong dipole on the NC-Mb could be interacted with the high dipole of the peptide bond on the protein surface. NC-Mb possesses a negative charge and is capable of adsorbing biomolecules with a positive charge (such as antibodies, antigens, or other proteins) through electrostatic adsorption [22]. An additional absorbance peak was observed on the FTIR after the urease enzyme was deposited (Figure 3I(d)). The reason for the broad peak at 3273 cm^−1^ is the stretching vibrations of the -OH and -NH_2_ that happen as a result of the amine group superimposing itself on the hydroxyl group band. The presence of the amino group of the urease enzyme was confirmed by the presence of this absorbance band [23]. The peak at 1546 cm^−1^ is related to the N-H in the amide group in the enzyme structure [24,25]. An additional amino group could indicate that there is a urease enzyme on the NC-Mb.

### 3.4. Optimization of the Urea Detection Using Urea-CLFS

#### 3.4.1. Effect of PR Concentration

**Figure 4:**
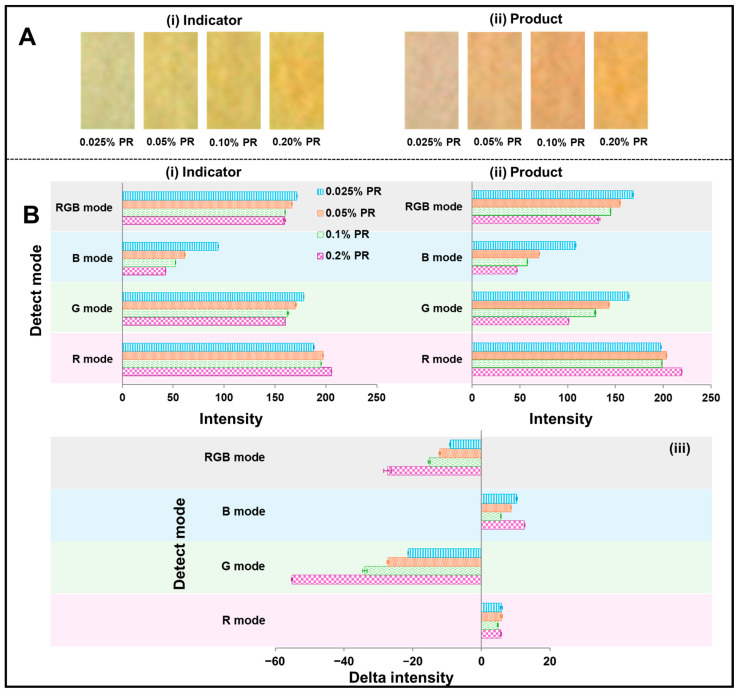
(**A**) The digital images of the reagent PAD on CH-PP before (**i**) and after (**ii**) tested with urea. (**B**) The intensity of reagent PAD before (**i**) and after (**ii**) tested with urea detected at 8 min reaction time and (**iii**) delta intensity of PR concentrations effect on the detection of urea.

The effect of PR concentration was studied, including 0.025, 0.05, 0.1, and 0.2% w/v. The solution was placed on CH-PP using a volume of 0.3 µL pipette and heated at 35 °C in the oven. Each condition was replicated three times. NC-Mb is utilized to hold urease (1000 U mL). The Biodot device dropped 20 dots of 5 nL each of urease onto the paper (0.1 U of urease enzyme total) and dried it using the same method as previously mentioned. The concentration of urea (30 mmol L^−1^) was formulated in DI water, and 12 µL was placed on the end of Urea-CLFS. The rapid scanning device was utilized to gauge the intensity of R, G, B, and RGB modes. The digital images are presented in Figure 4A(i,ii). The yellow color of the indicator became intense when the concentration of PR increased from 0.025 to 0.2% w/v. The rapid scanning device resulted in the RGB, G, and B intensities providing similar results with a decrease in intensity with PR concentration increasing (Figure 4B(i)), while the R mode does not provide a good trend for PR concentration. The intensity of RGB, G, and B modes shows a positive trend after urea was applied to the Urea-CLFS, with a decrease in intensity with PR concentration and fluctuation with R modes. Figure 4B(iii) displays the calculated delta intensity by dividing the product’s intensity (Figure 4B(ii)) by the indicator’s intensity (Figure 4B(i)). When the indicator reagent concentration increased, the delta intensity increased. Although 0.2% w/v PR yielded the highest signal, undissolved PR residue was occasionally observed at the bottom of the solution, suggesting limited solubility at this concentration. Therefore, 0.1% w/v PR was selected for subsequent experiments as it offered a strong and stable colorimetric response without solubility issues.

#### 3.4.2. Effect of Reagent Indicator Layer on NC Paper

**Figure 5:**
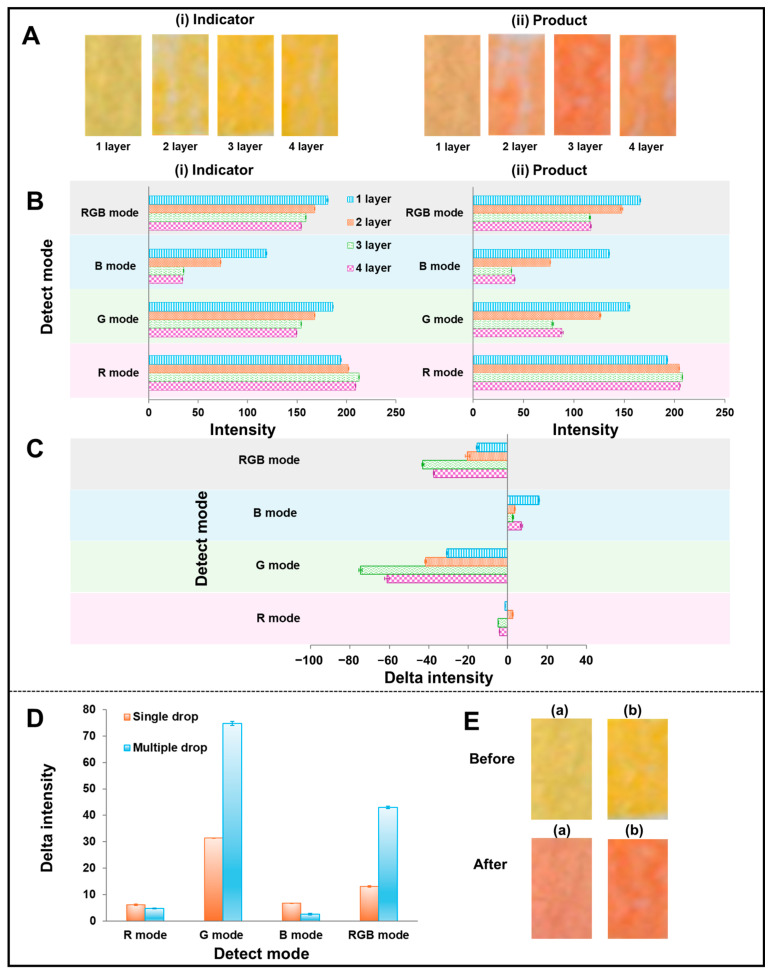
(**A**) The digital images of the reagent PAD on CH-PP before (**i**) and after (**ii**) tested with urea. (**B**) Comparison of color intensity before (**i**) and after (**ii**) tested with urea detected at 8 min reaction time for reagent PADs containing different numbers of phenol red indicator dispensing layers. Measurements were recorded across four detection modes (RGB, B, G, R modes). (**C**) The delta intensity of different dispensing layers of PR on the detection of urea. (**D**) The delta intensity of different preparations of reagent PAD with single and multiple drops. (**E**) The digital images of reagent PAD prepared by (**a**) single and (**b**) multiple drops of PR on CH-PP before and after testing with urea.

Thanks to its ability, the Biodot device can dispense a small nanoliter volume of solution on the substrate. In this way, the volume of reagent on the CH-PP can be dispensed and deposited in small quantities on layers of the reagent pad, and it can be studied. The preparation of reagent dispensing layers 1, 2, 3, and 4 is performed by dispensing 0.1 % w/v of the PR reagent indicator solution onto the CH-PP and using them for urea detection. Each layer was made by dispensing 5 nL of PR into six rows; each row consisted of 10 points (60 points × 5 nL = 300 nL). Each condition (different dispensing layers) was prepared and replicated three times. When the number of dispensing layers increased from one to three, the indicator’s color became intense as the digital images presented (Figure 5A(i,ii)). The intensity of four modes (RGB, B, G, R) correlated with the digital image, but the differences between the four dispensing layers and the three dispensing layers of the original indicator and color product were minimal (Figure 5B(i,ii)). After testing with urea, the delta intensity of RGB and G modes showed a similar pattern: the delta intensity increased from one to three dispensing layers and decreased to four dispensing layers (Figure 5C). Also, we evaluated the outcomes gathered by hand dropping with a micropipette (0.1 % w/v PR) in the previous experiment (Section 3.4.1 Effect of PR concentration). The clearer outcome was achieved with three layers. Therefore, three layers of reagent indicator were prepared using the Biodot device for urea detection in the next experiment.

To highlight the utilization of microdroplets by dispensing nanoliters on the substrate, the reagent PADs prepared by single and multiple drops using the same total volume of 0.9 µL were investigated. For multiple drops, 5 nL was administered to six rows, with each row having 10 dots with three dispensing layers, while single drops were prepared at one time with 0.9 µL. Each condition (both single and multiple drops) was replicated three times. Figure 5D shows that the delta intensity of urea detection obtained from reagent PAD prepared with multiple drops was higher than that obtained from reagent PAD prepared with a single drop. The digital images of the reagent/detection PAD obtained from multiple (Figure 5E(b)) and single drop (Figure 5E(a)) preparations in the situation before and after the test with urea are displayed in Figure 5E. When the RGB mode was considered, the intensity of reagent PAD made by multiple drops was roughly 3.3 times higher than that of a single drop. This result indicates that the way of reagent/detection PAD preparation is one of the crucial parameters to improve the sensitivity of Urea-CLFS.

#### 3.4.3. Optimized the Design of Urease Alignment on NC-Mb

**Figure 6:**
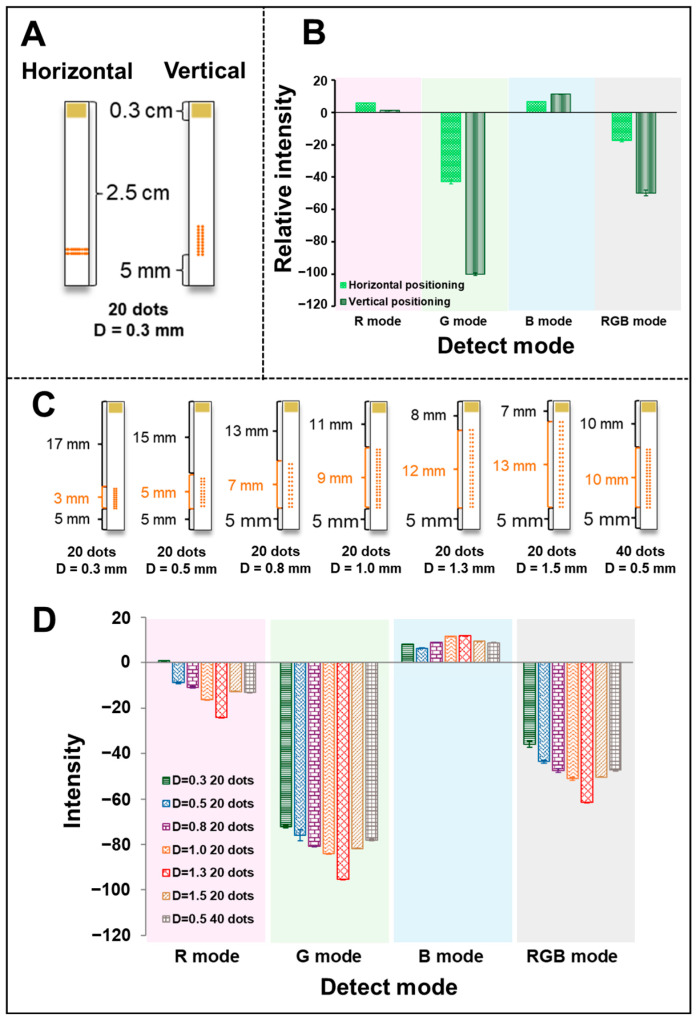
(**A**) The Urea-CLFS diagram images depicted the positioning of urease in both horizontal and vertical directions. (**B**) The histograms display the relative intensities of urea detection when the urease is located in different positions. (**C**) The diagram shows how to prepare Urea-CLFS using distances ranging from 0.3, 0.5, 0.8, 1.0, 1.3, and 1.5 mm. (**D**) The histograms of intensity of urea detection (30 mmol L^−1^) with different positioning of urease.

Our research led to the discovery of ways to align urease enzymes with the NC-Mb and modify its positioning to achieve the most effective urea detection performance. Figure 6A demonstrates how to apply urease to NC-Mb both vertically and horizontally using the same amount and number of dots (20 dots × 5 nL), and the same process was repeated for three PADs. Vertical positioning results in a higher intensity of color products for urea detection, as shown in the present result (Figure 6B). The reason is probably that the contact time between the urease enzyme and the sample is longer when positioning vertically compared to when positioning horizontally. Therefore, vertical positioning was selected for prepared urease on the NC-Mb. The discussion also included the distance (D) between each urease enzyme dot. Figure 6C illustrates the diagram of preparing Urea-CLFS using distances ranging from 0.3, 0.5, 0.8, 1.0, 1.3, and 1.5 mm, along with 20 dots (5 nL) of urease enzyme. The intensity of the color product was enhanced when the distance of enzyme dots alignment on the NC-Mb increased from 0.3 to 1.3 mm, but decreased after the distance was higher than 1.3 mm (Figure 6D). The urea detection intensity was the strongest when the distance between each enzyme dot was set to 1.3 mm. It was found that the enzyme’s catalytic performance is affected by its positioning, even though the amount of enzyme is equal. Consequently, further experiments were conducted with 1.3 mm of 5 nL each dot. To emphasize the impact of enzyme positioning, a double amount of urease enzyme was examined with the selected distance (D = 0.5 mm, 20 dots and D = 0.5 mm, 40 dots). The blue bar represents 20 dots, while the gray bar represents 40 dots using D = 0.5 mm. Even after double-folding the volume of the urease enzyme, the intensity of the color product (30 mmol L^−1^ urea) did not significantly increase, as shown in the results presented. Suitable positioning would lead to a reduction in urease enzyme consumption, as highlighted by the results. Consequently, the development of vertical alignment of the urease enzyme of the Urea-CLFSs can significantly enhance the performance of high-performance urea detection and for other screening devices as well.

#### 3.4.4. Effect of Urease Concentration and Reaction Time for the Enzymatic and Color Reaction

**Figure 7:**
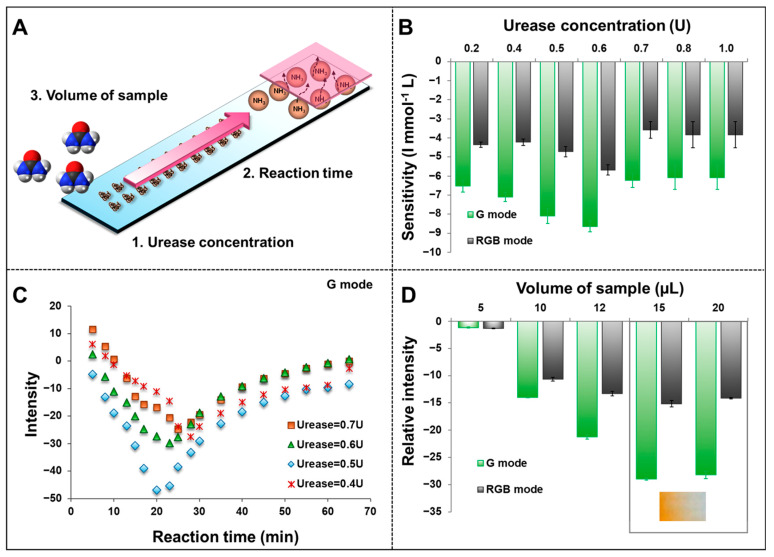
(**A**) The diagram shows the optimized parameter for urea detection based on the Urea-CLFS. (**B**) The histogram shows the sensitivity obtained from the urea detection concentrations of 2.5, 5.0, 7.5, and 10.0 mmol L^−1^. (**C**) The effect of reaction time on the detection of urea concentration of 2.5 mmol L^−1^ at different urease enzyme concentrations. (**D**) The relative intensity at different sample volumes on the detection of urea concentration of 2.5 mmol L^−1^.

To achieve the best performance of urea detection, the concentration of enzymes, detection time, and sample volume that are directly involved in enzymatic reactions were investigated (Figure 7A). The use of an enzyme allowed urease to convert urea to ammonia, which was then determined by Urea-CLFS. In this work, different concentrations of urease ranging from 0.1 to 1.0 U were tested with the fixed volume and numbers of urease amounts on NC-Mb. The sensitivity comparison of the urease concentration effect was performed by interpreting and utilizing the intensity of the different urea concentrations ranging from 2.5, 5.0, 7.5, and 10.0 mmol L^−1^ (three measurements for each). Figure 7B shows that the sensitivity of urea detection on G and RGB modes was enhanced when the concentration of urease increases from 0.1 to 0.6 U (Figure 7B). Afterwards, the sensitivity remained stable when using urease enzyme concentration of 0.7 to 1.0 U. To examine the effect of enzymatic reaction time on urea detection, urease concentrations of 0.4, 0.5, 0.6, and 0.7 U were employed in further research. The intensity of color development was measured every 5 min after applying the sample to the Urea-LFS to 65 min to investigate the trend. Figure 7C illustrates that the intensity of G mode gradually decreases as the enzymatic reaction time increases from the initial development of the color product. When the enzymatic reaction time reaches the highest point, the intensity tends to return to its original level since the product reaction was reduced by time. The intensity of urea detection is the biggest change when comparing 0.5 U on NC-Mb with different concentrations of urease enzymes. Therefore, the concentration of 0.5 U urease enzyme was selected for urea detection based on Urea-CLFS.

#### 3.4.5. Volume of Standard/Sample

The sample volume is crucial for analytical performance, as any change in sample volume will result in a change in the capillary flow rate, which has a significant impact on color development. Patients’ impact on testing can be minimized by lowering the number of samples collected. However, a small sample volume may lead to an increase in detection errors and a decrease in PAD’s linearity and sensitivity. Increasing sample volumes could cause bulk flow along the strip instead of capillary action, leading to a decrease in precision [26]. In order to accomplish this, it is necessary to analyze the precise sample volumes. Using a standard urea solution of 2.5 mmol L^−1^ (three measurements), the various liquid volumes, which included 5, 10, 12, 15, and 20 µL, were examined. Increasing the volume of fluid from 5 to 15 µL caused an increase in the intensity of color pads on reaction products, but they remained steady after 15 µL (Figure 7D). However, the color pad at 15 µL is sometimes not replicated because the color fades on the reagent pad (Figure 7D (inset)), causing the detection to not be replicated. It is probable that the amount of liquid exceeded the loading capacity of Urea-CLFS to hold it, and thus the liquid flowed out of the strip’s boundary and directly into the reagent PAD, as a result of irregular color development at the reagent PAD. In order to avoid this situation, therefore, 12 µL of liquid volume was selected for further experimentation.

### 3.5. Analytical Performance

**Figure 8:**
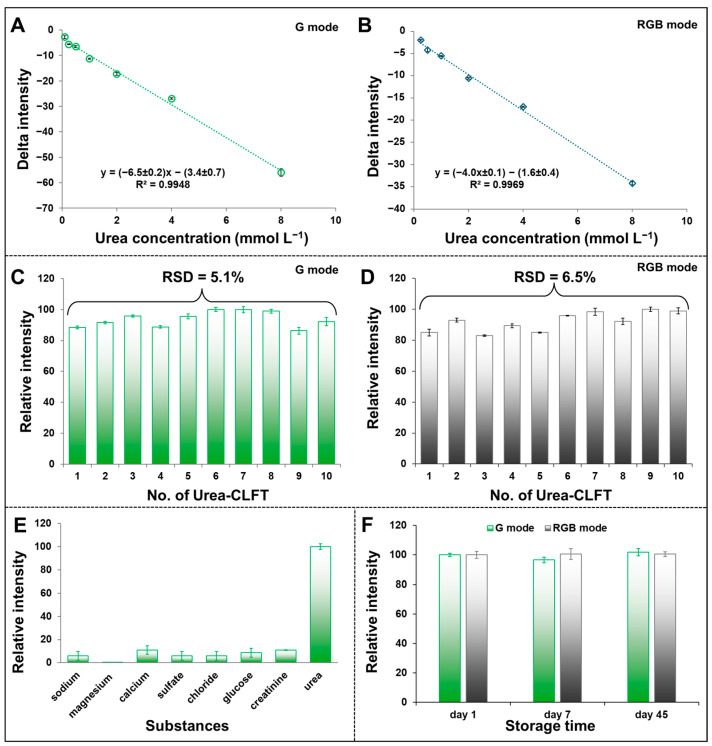
(**A**) Linear range of color intensity (G mode) response as a function of urea concentration. (**B**) Linear range of color intensity (RGB mode) response as a function of urea concentration. (**C**) Reproducibility of the Urea-CLFS at a urea concentration of 2.5 mmol L^−1^ detected in G mode. (**D**) Reproducibility of the Urea-CLFS at a urea concentration of 2.5 mmol L^−1^ detected in RGB mode. (**E**) Selectivity of the Urea-CLFS biosensor against several interferants. (**F**) Stability of sensor response over 45 days of storage at room temperature.

The analytical characteristics of the urea quantitative analysis with Urea-CLFS, such as linearity, precision, and accuracy, were investigated. To verify the detection sensitivity of Urea-CLFS, various concentrations of urea, including 0.25, 0.5, 1.0, 2.0, 4.0, and 8.0 mmol L^−1^, were tested with three measurements for each concentration. The G and RGB intensity was plotted against the urea concentration to compare the sensitivity response. At 25 min, the G intensity showed the highest sensitivity compared to the RGB mode. The linear equation was y = −6.5 ± 0.2x − 3.4 ± 0.7 with R^2^ = 0.9948 in the concentration range of 0.1 to 8.0 mmol L^−1^ (Figure 8A), while the RGB intensity provided the linear equation of y = −4.0 ± 0.1x − 1.6 ± 0.4, R^2^ = 0.9969 in the concentration range of 0.25 to 8.0 mmol L^−1^ (Figure 8B). To determine the limit of detection (LOD) of Urea-CLFS, three times the standard deviation of the y-intercept was divided by the slope of the calibration curve. In this case, the G mode was chosen, and the LOD was 0.34 mmol L^−1^, which is enough to detect urea in human urine and cover the urea concentration in blood. In order to assess the Urea-CLFS’s consistency, 10 individual sensors were exposed to 2.5 mmol L^−1^ of urea standard solution, with three measurements taken for each individual sensor. The determination’s relative standard deviation (RSD) was calculated once the results from G and RGB modes were received. The excellent reproducibility of urea colorimetric analysis can be explained by the low RSD obtained from the G and RGB mode, which was 5.1 and 6.5% (Figure 8C,D).

Following the creation and enhancement of the Urea-CLFS for urea detection, its qualities were contrasted with those described in other studies (Table 1). Compared to other methods, this device has detection limits that are similar to or even lower than those of other methods and has higher sensitivity when available, which is advantageous when analyzing lower urea concentrations. In terms of device preparation, this device necessitates less reagent and enzyme volume than most developed methods. Utilizing nanoliter volumes of reagents and enzymes could reduce chemical consumption and thus cost. Once all the reagents and samples are placed, the image and processing become even more complicated on another device [27,28,29,30,31]. For example, they need to take a photo in a specific condition and transfer the image to other software to evaluate the RGB intensity [28,30]. The Urea-CLFS has the capacity to accurately measure urea in human urine samples with an RSD lower than 5%, and it only requires buffer dilution; there is no need for acid or centrifugation. Furthermore, this work paves the way for the use of enzyme-based sensors using horizontal flow, which is achieved by separating catalysts and detection PADs. The horizontal flow is utilized to push the sample through capillary force with no external force.

The Urea-CLFS’s specificity for urea detection was examined. The common substances and compounds possibly existing in urine, including sodium (Na^+^), magnesium (Mg^2+^), calcium (Ca^2+^), sulfate (SO_4_^2−^), chloride (Cl^−^), glucose, and creatinine, were tested, taking three replicate measurements for each substance. As shown in Figure 8E, the delta intensity value for urea detection was much higher than that in the presence of non-target substances. It was demonstrated that the fabricated Urea-CLFS is able to detect urea with high selectivity. Additionally, Urea-CLFS’s storage stability was evaluated under room temperature storage conditions. The catalyst and detection PAD were prepared and kept in a sealed box placed at room temperature for different days. The stability of Urea-CLFS was evaluated by measuring the urea concentration of 2.5 mmol L^−1^ after several days, taking three replicate measurements on each day. The first day’s intensity value was taken into account to calculate the relative intensity. The Urea-CLFS’s performance was maintained during 45 days of storage at room temperature with lower RSD (<4%), as indicated by the result (Figure 8F). The results demonstrate that the Urea-CLFS exhibits excellent stability and can be utilized for at least 45 days after its preparation.

The detection of urea in human urine was investigated. Urine samples from healthy volunteers were used in this study. All donors gave verbal informed consent prior to sample collection. The samples were anonymized, and no personal or clinical data were obtained. The study was conducted in accordance with general ethical principles for non-interventional research. Samples 1 and 2 were diluted 100 times, while sample 3 was diluted 250 times with DI water before the measurement to match the detection range of the Urea-CLFS (0.1 to 8.0 mmol L^−1^). First, the intensity of the urine sample (with no spike) was recorded as described in Table S2. A paired *t*-test was used to evaluate the results obtained from both methods. At a 95% confidence level, it was observed that Urea-CLFS and the standard method have no significant differences between them (*t*calculated = 0.10, *t*critical = 2.91, *p* = 0.46 > *α* = 0.05), as indicated in the result. Therefore, Urea-CLFS is capable of quantifying urea in human urine samples. After spiking two concentrations of standard urea (10 and 25 mmol L^−1^) into the solution, the intensity of the result was analyzed. Using the calibration curve, the concentration of urea was calculated. The recovery values were calculated and are reported in Table S2. The recovery values ranged from 95 ± 3% to 103 ± 3% , which was acceptable under AOAC guidelines. Validity of the developed Urea-CLFS based on a biosensor is confirmed by the results.

## 4. Conclusions

This study is designed to introduce a new way of combining enzymes on NC-Mb and using detection PAD on different papers to enhance detection performance. Immobilizing a small volume of biomolecules, such as the urease enzyme, on NC-Mb was successful using the Biodot device. The CH-PP was utilized for the preparation of the detection PAD for holding phenol red reagent using the same method. The guidelines for different small volumes of reagent/sample were presented. The utilization of two paper types for distinct purposes was the primary element in achieving the colorimetric lateral flow strip for urea detection (Urea-CLFS). At the same volume, small-volume array dispensers provide a higher intensity change for urea detection compared to single drops. The Urea-CLFS revealed that the detection limit is 0.34 mmol L^−1^, which encompasses the urea concentration in blood and is more sufficient for urea in urine. The proposed colorimetric sensor design is effective in detecting urea in human urine, with results that are consistent with those of the standard method. By adding other paper, sample pads, and absorbance pads, the real sample analysis may offer direct urea detection in the real situation. Therefore, point-of-care screening of urea can be performed more easily and cost-effectively with the single-step colorimetric lateral flow strip.

While parameter optimization (e.g., enzyme loading, sample volume, and reaction time) significantly improved sensor performance, future enhancements could focus on increasing enzymatic efficiency. One promising strategy involves the use of enzyme–inorganic hybrid nanoflowers (HNFs), which are synthesized through co-precipitation using enzymes as templates to form inorganic nanostructures via self-assembly [33]. These nanoflowers offer superior catalytic activity and surface area compared to free enzymes, and their integration may further reduce reaction time and enhance signal output in paper-based biosensing platforms [34,35].

## Figures and Tables

**Table 1:** Colorimetric sensors based on lab-on-paper for enzymatic urea assay.

Approaches	Enzyme	Layers	Indicator	Sample Volume	Stability	Reaction Time	Analysis Device	Detection Mode	Sensitivity	Linear	LOD	Sample Preparation	Samples	Ref.
Horizontal flow (capillary force)														
Colorimetric lateral flow strip for urea detection (CLFS)	0.5 U (100 nL)	3 layers -Nitrocellulose membrane -Chromatography paper -Backing card	0.1% Phenol red (150 nL)	12 µL	45 days at RT *	25 min	RapidScan ST5-D	Green	−6.5 a.u. mmol^−1^ L	0.25 to 8.0 mmol L^−1^	0.34 mmol L^−1^	-	Urine	This work
Vertical flow (gravity force)														
Paper-based blood urea nitrogen optical biosensor	2 KU mL^−1^	4 layers -Mesh -Blood separation membrane -Filter film -Reaction film	0.12% Bromothymol blue	20 µL	-	2 min	Smartphone + portable optical reader	630 nm light source	6.6514 mmol^−1^ L RC **	2.46–38.14 mmol L^−1^	0.03 mmol L^−1^	Using separation membrane	Whole human blood	[29]
Smartphone-based optical biosensor	10 U	6 layers -Nylon mesh -Lamina films -Filter paper (Whatman number 1) -Card sheet	Phenol red	5 µL	30 days	20 s	Screenshot of the app + Smartphone based app using RGB profiling and slope-based calculation method	RGB	−0.005 average pixels sec^−1^/mgdL^−1^	100,000–2,600,000 mg L^−1^	104,000 mg L^−1^ (1.73 mol L^−1^)	-	Saliva	[27]
Microfluidic paper-based analytical device (micro-PAD) card	-	5 Layers -Laminate film -Whatman1 -PTFE hydrophobic membrane	0.8 mmol L^−1^ Bromothymol blue (15 µL)	12 µL	30 days at RT	5 min	Desktop scanner + image J	RGB	1.54 ± 0.06 × 10^−3^ Abs mg^−1^ L	15–50 mg L^−1^ 50–150 mg L^−1^ (0.25–2.50 mmol L^−1^)	11.3 mg L^−1^ (0.19 mmol L^−1^)	-	Saliva	[28]
Microfluidic paper-based analytical devices (μPADs)	1.875 U (15 µL)	6 Layers -Laminated film -Whatman4 -Whatman1 -Hydrophobic membrane	2 mmol L^−1^ Bromothymol blue (15 µL)	20 µL	30 days	35 min	Scan image + image J	RGB and Red	0.0823 a.u. mmol^−1^ L	0.163–1 mmol L^−1^ 1–5 mmol L^−1^	0.049 mmol L^−1^	-	Saliva	[30]
Paper-based colorimetric biosensor	12 mg mL^−1^ (150 µL)	-PVC paper card	Red cabbage extract	150 µL	10 days	30 min	Smartphone	RGB	−0.3827 a.u. mmol^−1^ L	0.5–100 mmol L^−1^	0.2 mmol L^−1^	-	Milk	[31]
Dip and read approach														
Paper strip	24 U mL^−1^	Filter paper (Whatman^®^ no. 3)	Bromothymol blue	20 mL	-	5 min	Take a photo and measure the color intensity by software	Blue	51.41 a.u. (%w/w)^−1^	0.10–1.0% (w/w)	-	Shake and sediment	Animal protein and fishmeal	[32]

* RT: room temperature. ** RC: reflection coefficient

## Data Availability

The original contributions presented in this study are included in the article/supplementary material. Further inquiries can be directed to the corresponding author.

## Supplementary Materials

The following supporting information can be downloaded at: https://www.mdpi.com/article/10.3390/bios15100688/s1, Table S1: The information of volume and open time used by the Biodot device; Table S2: Analytical performance of the Urea-CLFS for urea detection in spiked human urine samples compared to the standard method.
