# Supplementary material for: Nanoliter-Fabricated Paper-Based Colorimetric Lateral Flow Strip for Urea Detection
Source: Biosensors (Basel). 2025 Oct 11;15(10):688. doi: 10.3390/bios15100688 (PMC12562505; doi:10.3390/bios15100688)
Supplement: Supplementary file 1 [file biosensors-15-00688-s001.zip › biosensors-3794877-supplementary.pdf]

biosensors

MDPI

Supplementary materials

Nanoliter-Fabricated Paper-Based Colorimetric Lateral Flow Strip for Urea Detection

Supatinee Kongkaew [1,2,3], Suparat Cotchim [1,2,3] and Warakorn Limbut [1,2,3,4,*]

[1] Division of Health and Applied Sciences, Faculty of Science, Prince of Songkla University, Hat Yai, Songkhla, 90110, Thailand; supatinee.k@psu.ac.th (S.K.); suparat.c@psu.ac.th (S.C.)
[2] Center of Excellence for Trace Analysis and Biosensor, Prince of Songkla University, Hat Yai, Songkhla, 90110, Thailand.
[3] Center of Excellence for Innovation in Chemistry, Faculty of Science, Prince of Songkla University, Hat Yai, Songkhla,90110, Thailand.
[4] Forensic Science Innovation and Service Center, Prince of Songkla University, Hat Yai, Songkhla, 90110, Thailand.
* Correspondence: warakorn.l@psu.ac.th

Table S1. The information of volume and open time used by the Biodot device.

Dispensing volume (nL)	Recommendation open time range	Selected open time
5	50–150	100
10	100–300	200
20	200–600	400
30	300–900	600
40	400–1200	800
50	500–1500	1000
60	600–1800	1200
70	700–2100	1400
80	800–2400	1600
90	900–2700	1800
100	1000–3000	2000

Table S2. Analytical performance of the Urea-CLFS for urea detection in spiked human urine samples compared to the standard method.

Samples	Dilution factor	Standard method	Urea-CLFS	Urea-CLFS			
		Spike 0 mmol L^{-1}		Spike 10 mmol L^{-1} (pre-dilution)		Spike 25 mmol L^{-1} (pre-dilution)	
		Detected concentration (back-calculated to undiluted urine, mmol L^{-1}) (mean ± SD)		%Recovery (mean ± SD)	RSD (%)	%Recovery (mean ± SD)	RSD (%)
S1	100	85.92	92 ± 6	96 ± 4	3.5	100 ± 3	3.2
S2	100	93.34	93 ± 3	103 ± 3	2.8	98 ± 2	1.9
S3	250	188.95	182 ± 4	95 ± 3	4.6	98 ± 1	1.0

Note: Spike levels (10 and 25 mmol L^{-1}) refer to the pre-dilution additions. Spiking was performed before dilution. Detected concentrations are back-calculated to undiluted urine (measured × dilution factor). Validated linear range = 0.25–8.0 mmol L^{-1}.
